# Hemodynamic profiles of intubated and mechanically ventilated carbon monoxide-poisoned patients during systemic hyperbaric oxygen therapy

**DOI:** 10.1186/1471-2253-13-26

**Published:** 2013-09-27

**Authors:** Marie-Ludivine Chateau-Degat, Julien Poitras, Jacques H Abraini

**Affiliations:** 1 Hyperbaric Medicine and Gas Pharmacology Research Unit at the research center of the CSSS Alphonse Desjardins/CHAU de Lévis, Lévis, QC, Canada; 2 Family and Emergency Medicine Department, Faculty of Medicine, Université Laval, Québec, QC, Canada; 3 CHUQ Medical Research Center, Québec, QC, Canada; 4 Normandie Université, Université de Caen Basse Normandie, Caen, France; 5 Department of Anesthesiology, Faculty of Medicine, Laval University, Quebec, Canada

## Abstract

**Background:**

Carbon monoxide (CO) poisoning can be a life threatening condition. Systemic hyperbaric oxygen (HBO) therapy is used to induce CO detoxification. However, little is known about the hemodynamic response to HBO in severely intoxicated patients.

**Methods:**

We retrospectively analyzed the medical records of 6 CO-poisoned patients treated with propofol, rocuronium bromide, and HBO. The HBO protocol comprised 3 HBO treatments (HBOT1 to HBOT3) within 24 hours. During all HBO sessions heart rate (HR), systolic blood pressure (SBP), diastolic blood pressure (DBP), and pulse blood pressure (ΔBP) were measured every five minutes. Non-parametric tests were used to compare data between HBO sessions.

**Results:**

HR increased significantly as the number of HBOT increased, from 68 beats per minute (bpm) during HBOT1 to 77 and 86 bpm during HBOT2 and HBOT3, respectively (*p* < 0.05). In addition, while no significant change was found for DBP, both SBP and ΔBP showed a transient and significant increase during HBOT2, compared to HBOT1, that did not return to basal values during HBOT3.

**Conclusion:**

Based on previous studies that have established the respective effects of rocuronium bromide, propofol, HBO, and CO alone on HR, SBP, and ΔBP, it is concluded that the hemodynamic responses observed in the present study are likely to be due to CO. If such, given that neither HR nor SBP and ΔBP returned to basal values by the end of HBOT3, it is suggested that more than 3 HBOT sessions could be necessary to provide full hemodynamic recovery in CO-poisoned patients.

## Background

Carbon monoxide (CO) poisoning can be a life threatening condition that is associated with a long-term increased risk of mortality in severely intoxicated patients [1,2]. Because of the ability of oxygen to dissociate carboxyhemoglobin, high flow normobaric 100 vol% oxygen is used as a first-line therapy [3,4]. As another therapeutic strategy, systemic hyperbaric oxygen (HBO) therapy with 100 vol% oxygen is often used, when available, as a second line treatment in moderate to severe CO-poisoned patients to accelerate and improve the detoxification process [4-6]. However, although previous studies have established the effects of acute and repetitive HBO on the hemodynamic parameters of healthy subjects, certain types of patients, and laboratory animals [7-11], little is known on the hemodynamic effects of HBO in CO-poisoned patients. Thus, the purpose of this retrospective study was to assess the effects of HBO on the hemodynamic parameters of critically ill CO-poisoned patients.

## Methods

### Study design

This retrospective study is based on the review of the medical records of patients admitted for CO poisoning between 2008 and 2011 at the CSSS Alphonse-Desjardins Hôtel-Dieu de Lévis hospital (QC, Canada), and was approved by the CSSS Alphonse-Desjardins ethic committee with number # CER 1112–030.

### Patients and treatment

Among the 11 patients diagnosed for critical CO-poisoning based on their carboxyhemoglobin level upon arrival at the emergency department of CSSS Alphonse-Desjardins Hospital, 6 patients ≥ 18 years were included in the present study as they completed the entire drug treatment and HBO protocol described below. All patients were administered rocuronium bromide (Mckesson, Québec, Canada) to allow proper intubation before being placed in the pressure chamber, propofol (Mckesson, Québec, Canada) to provide sedation, and then were treated with HBO according to a protocol adapted from that of Weaver *et al*., [6]. This included 3 HBO treatments called HBOT1, HBOT2, and HBOT3 at a pressure of 2.5 to 2.8 atmospheres absolute (ATA) within 24 hours. During each HBOT, the patients were given 100 vol% oxygen for 3 periods of 30 minute duration as well as ambient air for 2 periods of 10 minute duration between each oxygen period. In-between the HBOTs, the sedated patients were brought back to the intensive care unit; there was no need for transportation of the sedated patients from the ICU to the pressure chamber in-between the HBO-treatments. All along their treatment in the pressure chamber and the intensive care unit, the patients were maintained intubated and sedated at a score of 5–6 on the Ramsay scale by administering rocuronium bromide and propofol repeatedly.

Based on clinical assessment, additional pharmacological treatments were given during the 24-h HBO/ICU period: Patient #3 was administered a single injection of 0.05 mg fentanyl before HBOT1 in addition of propofol; Patient #1 and Patient #5 were given ventolin®; Patient #1, Patient #3, and Patient #4 were given water-soluble vitamin B1 (thiamin). Also, Patient #3 and Patient #4 were given one additional HBOT. No catecholamine was given.

### Data collection & outcome measures

One investigator reviewed and abstracted the data from the medical records. Demographic information, CO-poisoning history, comorbidities, blood gas measurements, and sedation protocol were extracted. The patients’ hemodynamic profile comprising heart rate (HR), systolic blood pressure (SBP) and diastolic blood pressure (DBP) were also reviewed. Values measured from one of the patients’ arm using an Oscill Mate 1630 automated device (CASMED® Inc., Branford, CT, USA) were recorded every five minutes and averaged to obtain mean value during HBOT1, HBOT2, and HBOT3. Also, pulse blood pressure (ΔBP) was further assessed by calculating the difference between SBP and DBP.

### Data analysis

Data are expressed as the median value and the 25th and 75th percentiles, and analyzed using non-parametric methods with the SAS 9.2 software (SAS Institute, Cary, NC, USA). Within-group comparisons between HBOT were performed using the Friedman analysis of variance; following a significant *F* value, post hoc comparisons were made using the Wilcoxon *t*-test (two-tailed). The statistical significance was set at α = 5%.

## Results

Patients were 4 males and 2 females of 36 to 63 years of age. Four CO-poisonings were intentional and two were declared accidental. Three cases were related to fire smoke inhalation. Individual detailed demographic information, CO-poisoning history, and comorbidities are given in Table 1.

**Table 1:** Characteristics of patients upon arrival at the emergency department before first systemic hyperbaric oxygen treatment

**Patient**	**Age**	**Sex**	**CoHb**	**GSC**	**Tn1**	**Delay**	**Cardiovascular**	**ASA**
							**Comorbidities**	
#1	52	M	35%	4	0.02	5 h00	CAD	II
#2	36	M	37%	6	0.20	5 h45	No	I
#3	40	M	26%	9	0.53	5 h00	No	I
#4	48	M	55%	3	0.52	5 h00	SSS/PM	IV
#5	64	F	50%	3	0.08	3 h45	T2DM	IV
#6	63	F	34%	15	0.06	12 h00	HTN	III

Values collected in the emergency room before the patient was intubated and then treated with hyperbaric oxygen (HBOT1); GSC: Glasgow Coma Scale upon arrival at the emergency department; CoHb: Carboxyhemoglobin (values recorded before arrival at CSSS Alphonse-Desjardins Hôtel-Dieu de Lévis hospital); Tn1: troponin 1; Delay: between first primary care by paramedics and HBOT1; CAD: Coronary artery disease; SSS/PM: Sick sinus syndrome/pacemaker; T2DM: Type 2 diabetes mellitus; HTN: Hypertension. ASA: American Society of Anesthesiology patient classification status [12]. ASA-I: completely healthy fit patient; ASAII: Patient has mild systemic disease; ASA-III: severe systemic disease that is not incapacitating; ASA-IV: Patient has incapacitating disease that is a constant threat to life; ASA-V: A moribund patient who is not expected to live 24 hour with or without surgery.

We examined the effects of HBOT on the hemodynamic parameters of these CO-poisoned patients. We found that HR increased significantly as the number of HBOT increased from 68 beats per minute (bpm) during HBOT1 to 77 and 86 bpm during HBOT2 and HBOT3, respectively (F = 7, *p* < 0.05). This resulted in a trend toward increase in HR between HBOT1 and HBOT2 (T = 1.892, *p* < 0.1) that reached statistical significance between HBOT1 and HBOT3 (T = 2.207, *p* < 0.05). SBP showed significant changes across treatments from 102 mmHg during HBOT1 to 130 and 112 mmHg during HBOT2 and HBOT3, respectively (F = 8.333, *p* < 0.02). This led to a significant difference in SBP between HBOT1 and HBOT2 (T = 2.201, *p* < 0.05) but not between HBOT1 and HBOT3 (T = 1.153). In addition, ΔBP also showed significant changes across treatments from 44 mmHg during HBOT1 to 58 and 49 mmHg during HBOT2 and HBOT3, respectively (F = 9.250, p = 0.01). This resulted in a significant difference in ΔBP between HBOT1 and HBOT2 (T = 2.214, *p* < 0.05), but not between HBOT1 and HBOT3 (T = 1.577). In contrast with HR, DBP and ΔBP, no significant change was found for DBP (F = 0.882). Individual data of HR, SBP, DBP, and ΔBP are shown in Table 2.

**Table 2:** Hemodynamic profile through the HBO treatments

	**HR**			**SBP**			**DBP**			**ΔBP**		
	**(beats per min)**			**(mmHg)**			**(mmHg)**			**(mmHg)**		
**Patient**	**HBOT1**	**HBOT2**	**HBOT3**	**HBOT1**	**HBOT2**	**HBOT3**	**HBOT1**	**HBOT2**	**HBOT3**	**HBOT1**	**HBOT2**	**HBOT3**
#1	70	73	75	101	129	142	61	74	75	40	54	66
#2	63	76	66	93	114	108	56	60	59	37	54	49
#3	71	68	74	99	113	109	60	59	59	39	54	49
#4	87	103	97	104	134	115	55	68	66	49	66	48
#5	67	78	100	125	131	107	76	69	59	49	62	48
#6	61	83	111	134	147	143	67	65	69	67	82	74
Median	69	77	86*^†^	103	130*	**112**^**†**^	61	67	63	45	58*	**49**^**†**^
Q1	64	74	74	100	118	108	57	61	59	39	54	48
Q3	71	82	99	120	133	135	66	69	68	49	65	62

Individual data, and median and quartiles values of heart rate (HR), systolic blood pressure (SBP), diastolic blood pressure (DBP) and pulse blood pressure (ΔBP) during HBOT1, HBOT2, and HBOT3. Patients #1 and #6 suffered coronary artery disease and hypertension, respectively. * : *p* < 0.05 *vs* HBOT1. ^†^ : *p* = n.s. *vs* HBOT2, indicating so far as SBP and ΔBP are concerned that HBOT3 values had not return to HBOT1 values. Individual data of HR, SBP, and DBP were obtained by calculating the mean of the 26 values recorded every 5 minutes during HBOT1, HBOT2, and HBOT3.

## Discussion and conclusions

In this retrospective study performed in critically ill CO-poisoned patients treated with rocuronium bromide, propofol, and HBO, we observed both a sustained increase in HR and a transient increase in SBP and ΔBP.

In contrast with these effects, previous hemodynamic anesthesia studies have reported no or little vagolytic effects of rocuronium bromide alone on HR, SBP, and DPB [13-15]. Also, in contrast with these effects and the findings of the present report, propofol has been demonstrated in other anesthesia studies to produce marked decreases in HR, SBP and DBP [16-18]. Likewise, HBO studies in healthy subjects, non-CO-poisoned patients and laboratory animals have also reported marked decreases in HR [7-11], SBP and DBP [7], as a consequence of hyperbaric oxygen rather than increased pressure *per se*[7]. In contrast with these effects of rocuronium bromide, propofol and HBO, acute CO-poisoning with carboxyhemoglobin values above 25% has been commonly reported to increase HR [19,20] as well as, in a more controversial fashion, both SBP and ΔBP [20]. Taken together, these data suggest that CO could appear as a good candidate to explain our findings; however, why these CO effects, if such, increased across treatments i.e. showed long-lasting effects despite HBO therapy is a question that still remains to be elucidated. Based on a previous study that has reported that most of the myocardial dysfunction as evaluated using cardiac biomarkers and ejection fraction measurements (but not hemodynamic parameters) dissipates at 24 hours in CO-poisoned patients [21], it could be hypothesized that adverse interactions between rocuronium bromide, propofol, HBO, and/or CO could be responsible for the increase in HR, SBP, and ΔBP observed in the present study. However, the individual effects of rocuronium bromide, propofol, and HBO – which all decrease HR, SBP and DBP when given alone – clearly question such a possibility. With no doubt, only a randomized controlled trial adequately designed would be able to identify the actual contribution, if any, of rocuronium bromide, propofol, and/or HBO in the results observed.

Thus, if one assumes that the sustained increase in HR from HBOT1 to HBOT3 as well as the transient increase in SBP and ΔBP from HBOT1 to HBOT2 reported herein are the consequence of CO poisoning, then the decrease in SBP and ΔBP recorded between HBOT2 and HBOT3 could be viewed as a beneficial effect of HBO, which after detoxifying hemoglobin could allow initiating the detoxification of other hemoproteins such as myoglobin whose normal functioning is known to be necessary for effective cardiac output. However, although both SBP and ΔBP showed a general trend toward reduction between HBOT2 and HBOT3, which could indicate as suggested above that HBO had begun to produce its beneficial effects, a careful examination of the patients’ hemodynamic responses revealed that 2 subjects with cardiovascular comorbidities (hypertension and coronary artery disease) still exhibited borderline hemodynamic responses [22-27], with a SBP increase above 140 mmHg and a ΔBP increase near or above 80 mmHg. Taken together with the sustained increase in HR recorded from HBOT1 to HBOT3, these data suggest that the detoxification of myoglobin by HBO could be longer than generally thought, and that more 3 HBOT sessions could be necessary to allow full hemodynamic recovery in CO-poisoned patients or at least some of them.

As the vast majority of the retrospective case-series studies, the present report should be interpreted carefully because of its inherited limitations. This includes the small sample of patients, the lack of information on the duration of the exposure to CO, the uncontrolled delay between the end of the exposure to CO and the first HBOT session, the uncontrolled administration of medication, and the absence of actual post-treatment evaluations that could have indicated that HR, SBP, and ΔBP had finally returned to basal values. However, despite these limitations, we believe that the present study is of actual interest since it is the first one, to the best of our knowledge, to report the hemodynamic effects of HBO in critically ill CO-poisoned patients.

Given the respective effects of rocuronium bromide, propofol, HBO, and CO on the hemodynamic parameters, we conclude as discussed in details above that the increase in HR, SBP and ΔBP observed in the present study is likely to be due to CO, and that more than 3 HBO sessions would be necessary to provide full hemodynamic recovery in CO-poisoned patients. If such, it is likely that HR, SBP, and ΔBP could be used as physiological markers to assess CO detoxification. Monitoring these hemodynamic parameters together with patient outcomes in future prospective clinical studies could document this possibility. With no doubt, further studies are needed to confirm our hypothesis, and lead clinicians to use hemodynamic parameters as a clinical biomarker for CO-poisoning and HBO detoxification.

## Consent

Written informed consent was obtained from the representant of the participant for the publication of this report.

## Competing interests

The authors declare that they have no competing interests.

## Authors’ contributions

JP conceived the study and actively participated in the data collection. MLCD and JHA designed the study, analyzed the data and drafted the manuscript. All of the authors interpreted, revised and approved the final version of the manuscript for publication. MLCD takes the responsibility for this paper as a whole.

## Pre-publication history

The pre-publication history for this paper can be accessed here:

http://www.biomedcentral.com/1471-2253/13/26/prepub
